# Crystal structure of tris­[4-(3,4-di­meth­oxy­thio­phen-2-yl)phen­yl]amine

**DOI:** 10.1107/S2056989026000058

**Published:** 2026-01-08

**Authors:** Masafumi Yano, Yukiyasu Kashiwagi, Koki Oishi, Minori Yano, Koichi Mitsudo

**Affiliations:** a Kansai University, 3-3-35 Yamate-cho Suita Osaka 564-8680 Japan; b Osaka Research Institute of Industrial Science and Technology, 1-6-50 Morinomiya Joto-ku Osaka 536-8553 Japan; c Okayama University, 3-1-1 Tsushima-naka, Kita-ku, Okayama 700-8530, Japan; University of Hyogo, Japan

**Keywords:** crystal structure, infrared absorption dye, one-electron oxidation

## Abstract

In the title compound tris­[4-(3,4-di­meth­oxy­thio­phen-2-yl)phen­yl]amine (DMOT-TPA), C_36_H_33_NO_6_S_3_, the central nitro­gen atom shows no pyramidalization, with the three *para*-phenyl­ene rings arranged in a propeller-like geometry. Each thio­phene ring is twisted by about 25–29° relative to the adjacent phenyl­ene ring, giving a distorted π-conjugated framework. In the crystal, mol­ecules are linked through multiple C—H⋯π inter­actions into two-dimensional sheets, which extend into a three-dimensional network. A Cambridge Structural Database survey revealed no prior examples of tri­phenyl­amines bearing 3,4-di­meth­oxy­thio­phen units at the *para* positions. This unique structure provides new insights into the design of redox-active organic materials.

## Abstract 2

The crystal structure of tris­[4-(3,4-di­meth­oxy­thio­phen-2-yl)phen­yl]amine (DMOT-TPA) was determined by X-ray diffraction. The central nitro­gen atom is non-pyramidal, with the three *para*-phenyl­ene rings in a propeller arrangement. The thio­phene rings are twisted by *ca*. 25–29° relative to the phenyl­ene rings, forming a distorted π-conjugated framework. In the crystal, C—H⋯π inter­actions link mol­ecules into two-dimensional sheets and a three-dimensional network.

## 1. Chemical context

Tri­aryl­amines (TAAs) are well-known electron donors and continue to be the subject of much theoretical and experimental research. Since TAA derivatives with various substituents in the *para*-position give stable radical cations by one-electron oxidation in solution, they are used in the fields of positively charged purely organic high-spin systems (Sato *et al.*, 1997 ▸) as well as for organic mixed-valence mol­ecular systems (Lambert *et al.*, 1999 ▸). TAAs with extra aromatic rings in the *para*-position have received notable attention as components of redox-active organic materials (Yen & Liou, 2012 ▸; Thelakkat, 2002 ▸). Among them, tris­(4-(thio­phene-2-yl)phen­yl)amine and its π-extended derivatives have been developed into electroactive polymer electrodes and electrochromic polymer materials, and many derivatives continue to be reported (Golba *et al.*, 2015 ▸). Thio­phenes with strong electron-donating substituents at the β-position show enhanced donor properties and high stability. Consequently, a tri­phenyl­amine derivative incorporating three 3,4-di­meth­oxy­thio­phene groups is expected to behave as a redox-active core that enables facile electron transfer in both solution and the solid state, with potential relevance to mol­ecular electronic materials. We report herein on the crystal structure of the title compound.
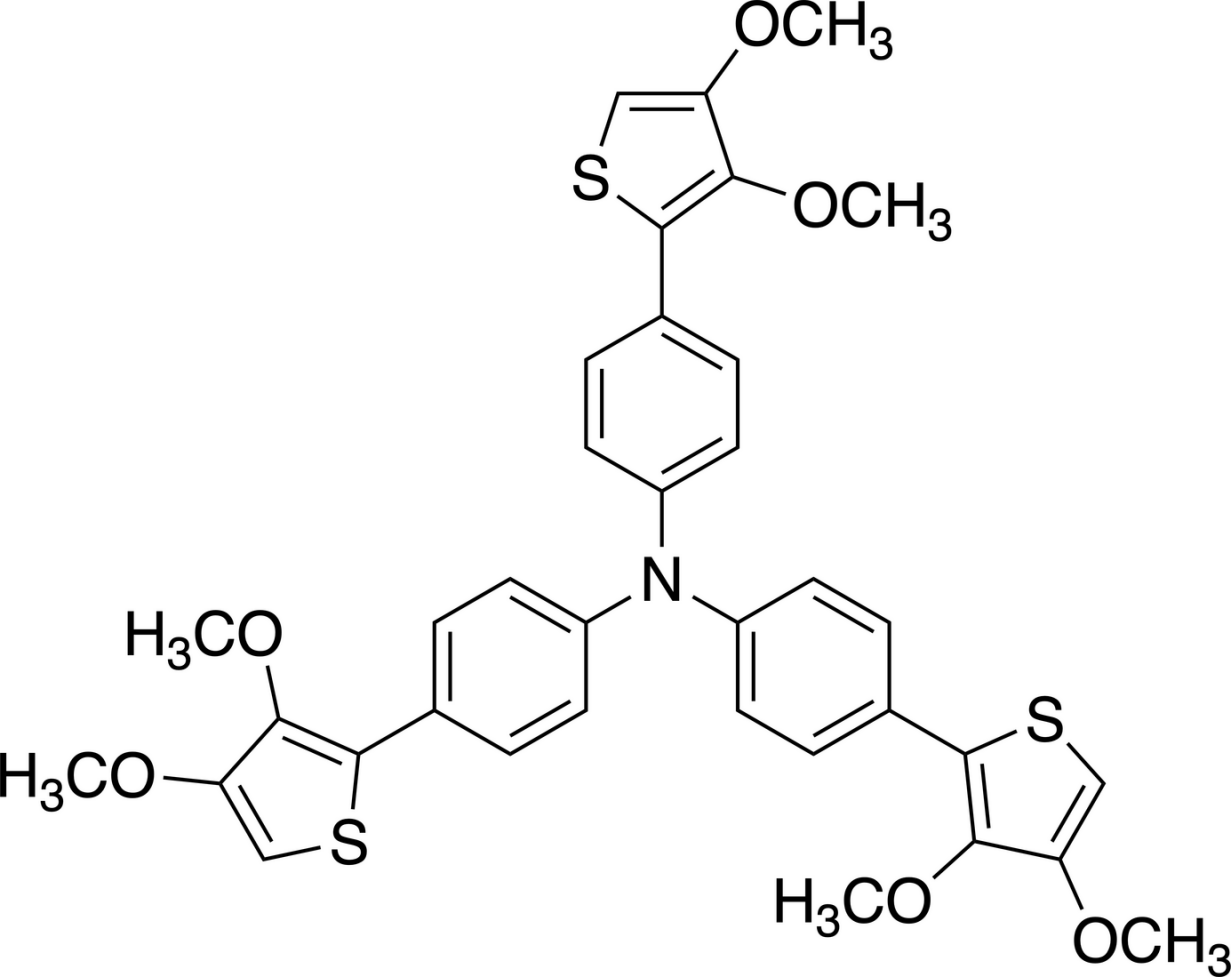


## 2. Structural commentary

**Figure 1:**
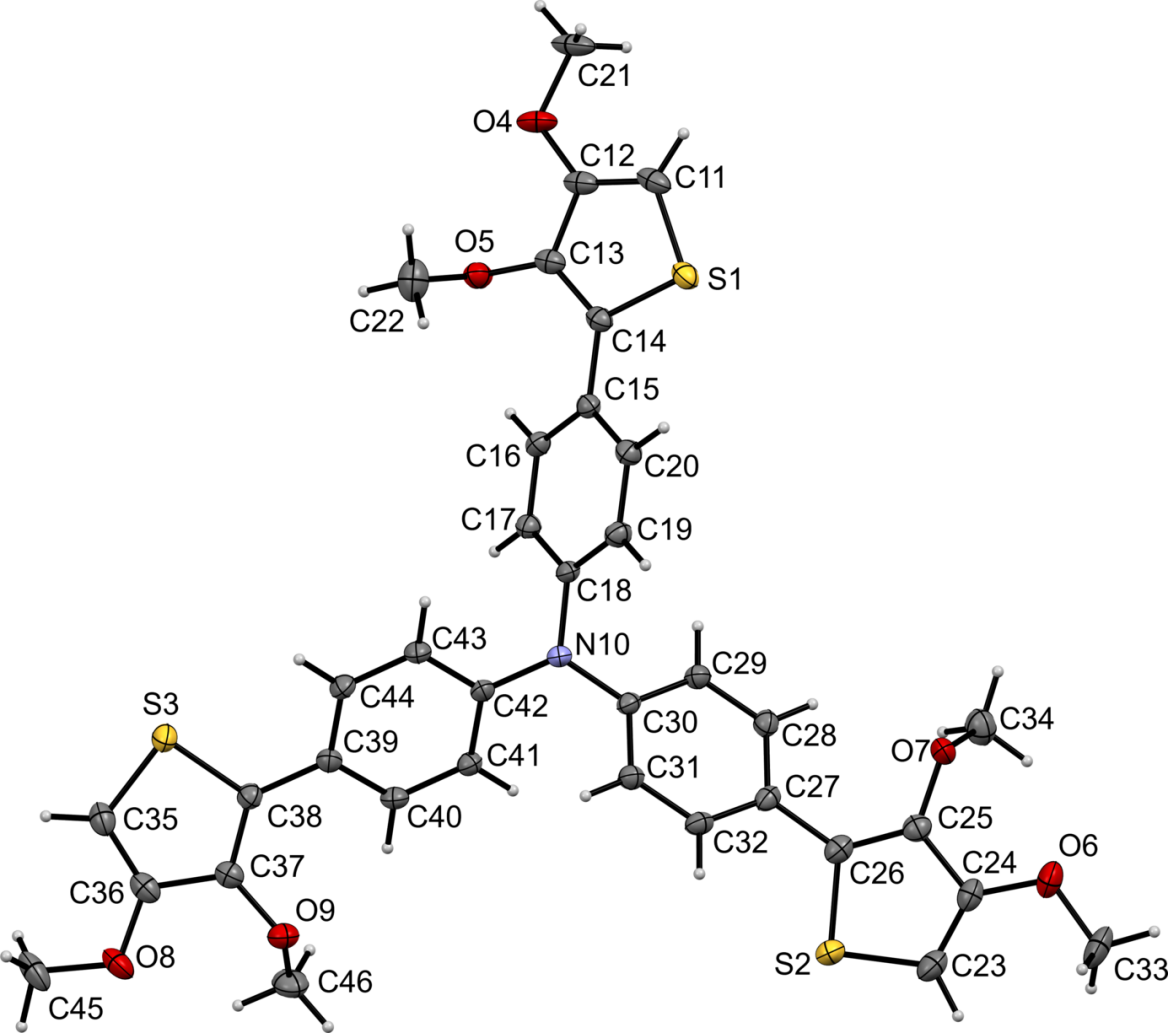
The mol­ecular structure of the title compound with the atom labeling. Displacement ellipsoids are drawn at the 50% probability level. H atoms are represented by spheres of arbitrary radius.

The mol­ecular structure of the title compound is shown in Fig. 1 ▸. The central N10 atom shows no pyramidalization, with a deviation from the plane of the bonded C atoms (C18, C30, and C42) of 0.025 (2) Å. The three *para*-phenyl­ene rings are bonded to the N10 atom in a propeller-type fashion, which is a common arrangement for Ph_3_N fragments. The torsion angles C17—C18—N10—C42, C29—C30—N10—C18 and C41—C42—N10—C30 are −49.7 (2), −30.3 (2) and −29.8 (2)°, respectively. The mean planes of the *para*-phenyl­ene rings and the neighboring thio­phene ring are inclined to each other by 24.19 (10)° for (C15–C20)/(S1/C11–C14), 28.73 (9)° for (C27–C32)/(S2/C23–C26) and 26.67 (9)° for (C39–C44)/(S3/C35–C38).

## 3. Supra­molecular features

**Figure 2:**
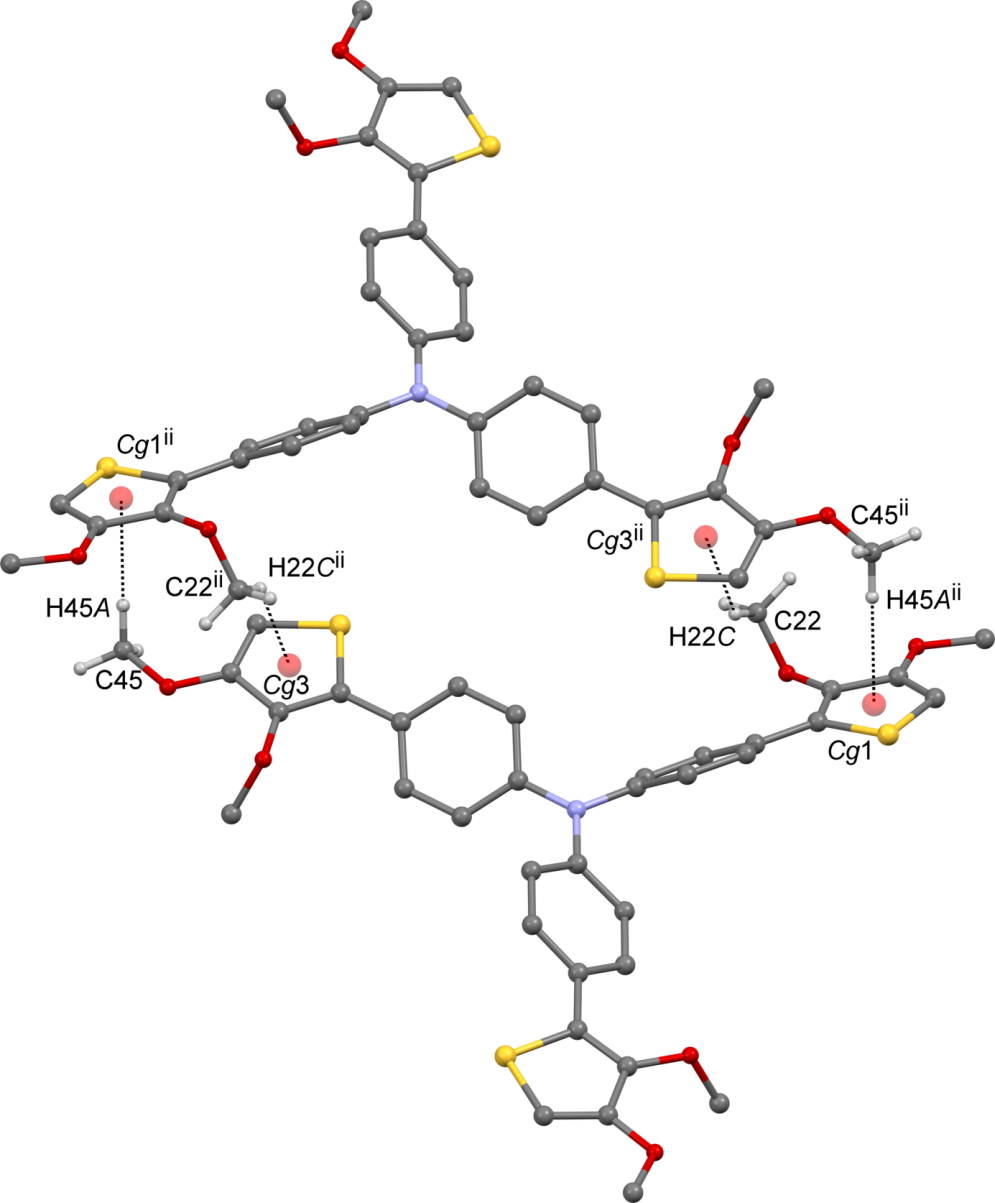
The centrosymmetric dimeric structure of the title compound. The inter­molecular C—H⋯*π* inter­actions are shown as dashed lines. H atoms not involved in these inter­actions have been omitted for clarity. [Symmetry code: (ii) −*x* + 1, −*y* + 1, −*z* + 1.]

**Figure 3:**
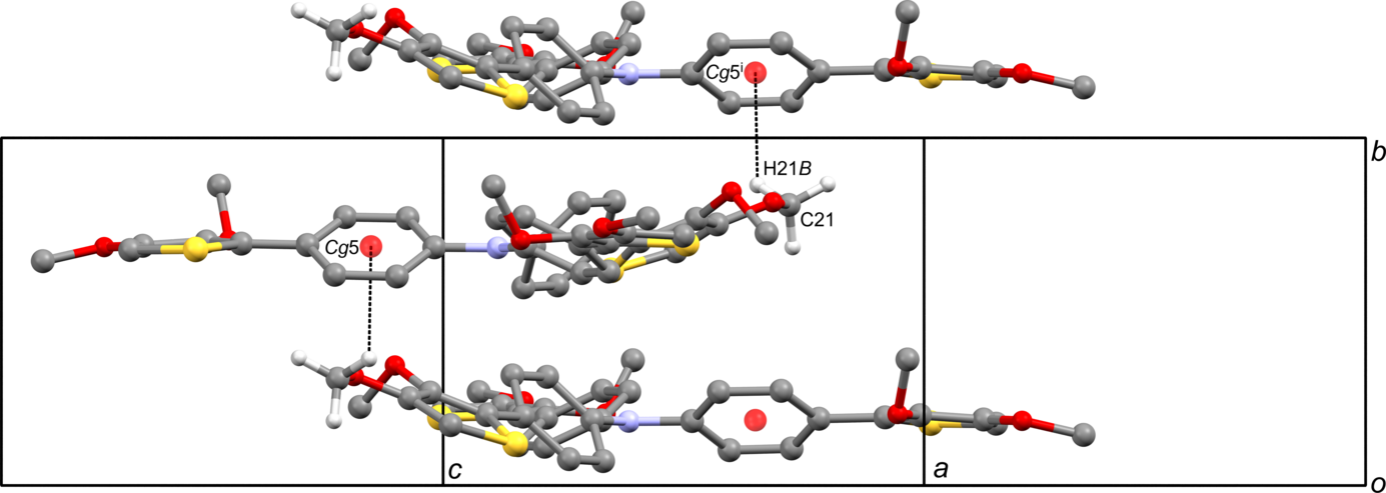
A portion of the crystal packing of the title compound showing the spiral chain formed *via* a 2_1_ screw axis. The inter­molecular C—H⋯*π* inter­actions are shown as dashed lines. H atoms not involved in these inter­actions have been omitted for clarity. [Symmetry code: (i) −*x* + 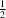, *y* + 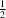, −*z* + 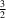.]

In the crystal, each mol­ecule inter­acts with five others via four inter­molecular C—H⋯*π* inter­actions (Table 1 ▸). The mol­ecules are linked by complementary C—H⋯*π* inter­actions between the meth­oxy group and a neighboring thio­phene ring [C22—H22*C*⋯*Cg*3^ii^ and C45—H45*A*⋯*Cg*1^ii^; *Cg*3 and *Cg*1 are the centroids of the S3/C35–C38 and S1/C11–C14 rings, respectively; symmetry code: (ii) −*x* + 1, −*y* + 1, −*z* + 1], forming an inversion dimer (Fig. 2 ▸). The other two C—H⋯*π* inter­actions [C21—H21*B*⋯*Cg*5^i^ and C32—H32⋯*Cg*5^iii^; *Cg*5 is the centroid of the C27–C32 ring; symmetry code: (i) −*x* + 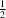, *y* + 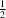, −*z* + 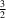; (iii) −*x* + 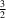, *y* − 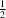, −*z* + 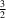] form one-dimensional chain structures parallel to the *b*-axis (Figs. 3 ▸ and 4 ▸), and these inter­actions form two-dimensional sheets in the *ac* plane. As a result, the two-dimensional sheets are linked by complementary C—H⋯π inter­actions, forming the dimers mentioned above into a three-dimensional network. Weak inter­molecular inter­actions [O5⋯H45*C*—H45^iv^ and O9⋯H34*B*—C34^iii^; symmetry code: (iv) −*x* + 1, −*y* + 2, −*z* + 1] are also shown in Table 1 ▸. There are no significant inter­molecular inter­actions around *Cg*2, *Cg*4 and *Cg*6 (the centroids of the S2/C23–C26, C15–C20 and C39–C44 rings, respectively).

**Figure 5:**
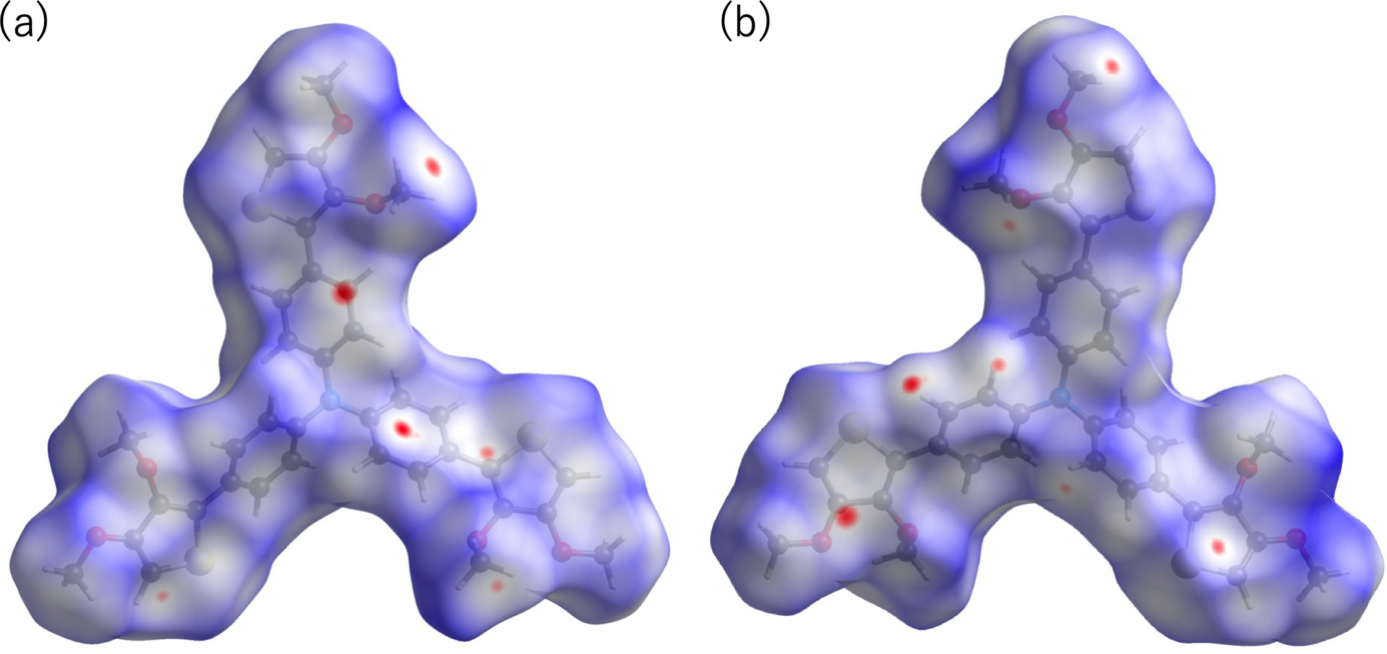
Hirshfeld surface mapped over *d*_norm_ for the title compound. (*a*) Front view and (*b*) back view. Red spots indicate short C—H⋯O and C—H⋯S contacts.

**Figure 6:**
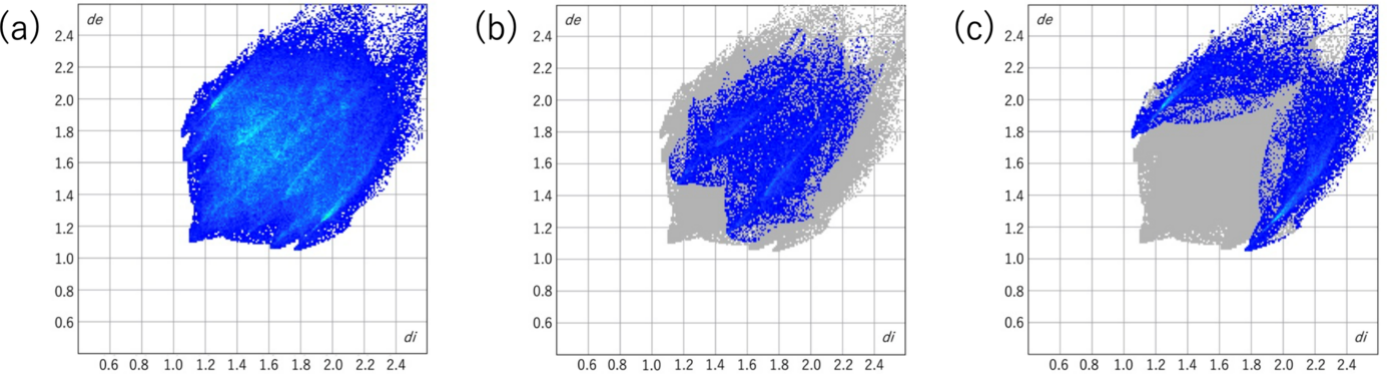
Two-dimensional fingerprint plots for the title compound. (*a*) Full fingerprint plot showing the overall distribution of *d*_i_ and *d*_e_. Fingerprint plots highlighting the (*b*) H⋯O/O⋯H contacts and (*c*) H⋯S/S⋯H contacts.

In order to further characterize the inter­molecular inter­actions in the crystal of the title compound, a Hirshfeld surface analysis (Spackman & Jayatilaka, 2009 ▸) was carried out using *CrystalExplorer* (version 21.3; Spackman *et al.*, 2021 ▸). The Hirshfeld surface mapped over *d*_norm_ (Fig. 5 ▸) shows several localized red spots, which correspond to short C—H⋯O and C—H⋯S contacts between neighboring mol­ecules. The associated two-dimensional fingerprint plots (McKinnon *et al.*, 2007 ▸) provide qu­anti­tative information on the inter­molecular inter­actions in terms of percentage contributions (Spackman & McKinnon, 2002 ▸). As illustrated in Fig. 6 ▸, H⋯H contacts contribute 46.8% to the Hirshfeld surface and dominate the crystal packing, followed by H⋯O/O⋯H (12.7%) and H⋯S/S⋯H (12.8%) contacts.

## 4. Database survey

A search of the Cambridge Structural Database (CSD, Version 6.00, update August 2025; Groom *et al.*, 2016 ▸) for compounds containing tri­phenyl­amines yielded 9691 hits (including 8428 hits for non-polymeric compounds). Limiting the search to non-polymeric tri­phenyl­amines with at least one thio­phene ring bonded to tri­phenyl­amine core at the 4-position of phenyl group gave 167 hits (149 compounds), which included five hits (five compounds) with the thio­phene ring having oxygen atoms at the two *β*-positions. There was one report of TPA cores bound to EDOT units (BUSZIC; Yuan *et al.*, 2021 ▸). There are five compounds with thio­phene rings at the three *para-*positions of tri­phenyl­amine, three of which do not contain metal ions: tris­(2-thio­phenyl-4-phen­yl)amine (AXELIZ; Wang *et al.*, 2011 ▸), its tri­formyl­ated compound tris­[2-(5-formyl­thio­phen­yl)-4-phenl­yl]amine (PAYXAQ; Par­thasarathy *et al.*, 2011 ▸) and tris­(2-{5-[*N*-(butan-2-yl)-2-cyano­prop-2-enamide]­thio­phen­yl}-4-phenl­yl)amine (SODXOB; Adelizzi *et al.*, 2019 ▸). These three compounds adopt a similar propeller-type TPA geometry. A search for compounds containing 2,3-di­meth­oxy­thio­phene yielded nine hits of which six are non-macrocyclic compounds. Of these six compounds, only two structures contain an aryl-substituent at the *α*-position of thio­phene [ILIWAF (Peng *et al.*, 2025*a* ▸) and ILIWEJ Peng *et al.*, 2025*b* ▸)]. The inter­molecular inter­actions around 2,3-di­meth­oxy­thio­phene were not shown in ILIWEJ, whereas in contrast only a thio­phenyl S⋯H—N inter­action was suggested in ILIWAF.

## 5. Synthesis and crystallization

DMOT-TPA was synthesized under Negishi coupling conditions using the method we previously reported (Yano *et al.*, 2022 ▸). To a solution of 3,4-di­meth­oxy­thio­phene (0.10 mL, 0.91 mmol) in tetra­hydro­furan (THF, 1.20 mL) were added 1.6 *M* of *n*-BuLi in hexane (0.60 mL, 0.96 mmol) at 195 K. After stirring at 195 K for 1 h, 1.0 *M* of ZnCl_2_ in THF (0.96 mL, 0.96 mmol) was slowly added and stirred for 0.5 h at 273 K. 4,4′,4′′-Tri­bromo­tri­phenyl­amine (0.11 g, 0.22 mmol) and tris­(di­benzyl­ideneacetone)dipalladium(0)·CHCl_3_ (3.4 mg, 0.006 mmol), 2-di­cyclo­hexyl­phosphino-2′,6′-di­meth­oxy­biphenyl (SPhos, 8.5 mg, 0.020 mmol) were added and stirred at 343 K for 1 h. The resulting solution was quenched with water, extracted with chloro­form, and dried over sodium sulfate. Upon addition of an excess amount of methanol to this solution, a yellow powder was precipitated (111 mg, 76%). ^1^H NMR (400 MHz, CDCl_3_): *δ* 3.84 (*s*, 9H), 3.86 (*s*, 9H), 6.10 (*s*, 3H), 7.12 (*d*, *J* = 8.8 Hz, 6H), 7.60 (*d*, *J* = 8.8 Hz, 6H). Pale-yellow crystals of DMOT-TPA suitable for X-ray diffraction were obtained by slowly evaporating a solution dissolved in a mixture of aceto­nitrile and toluene.

## 6. Refinement

Crystal data, data collection and structure refinement details are summarized in Table 2 ▸. The C-bound H atoms were placed in geometrically calculated positions (C—H = 0.95–0.99 Å) and were constrained using a riding model with *U*_iso_(H) = 1.2 *U*_eq_(C) for aromatic H atoms and *U*_iso_(H) = 1.5*U*_eq_(C) for methyl H atoms. Anisotropic displacement parameters for the C25 and O7 were refined with enhanced rigid bond (RIGU) restraints.

## Supplementary Material

Crystal structure: contains datablock(s) I. DOI: 10.1107/S2056989026000058/ox2019sup1.cif

Structure factors: contains datablock(s) I. DOI: 10.1107/S2056989026000058/ox2019Isup3.hkl

CCDC reference: 2520149

Additional supporting information:  crystallographic information; 3D view; checkCIF report

## Figures and Tables

**Figure 4:**
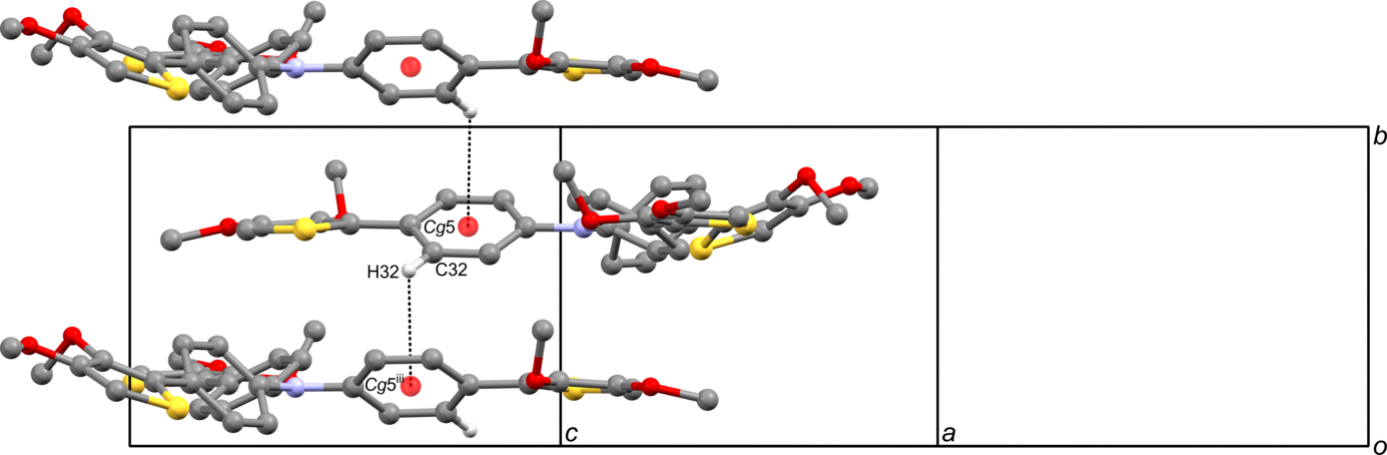
A portion of the crystal packing of the title compound showing the spiral chain formed *via* a 2_1_ screw axis. The inter­molecular C—H⋯π inter­actions are shown as dashed lines. H atoms not involved in these inter­actions have been omitted for clarity. [Symmetry code: (iii) −*x* + 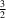, *y* − 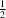, −*z* + 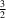.]

**Table 1:** Hydrogen-bond geometry (Å, °) *Cg*1, *Cg*3 and *Cg*5 are the centroids of the S1/C11–C14,S3/C35–C38 and C27–C32 rings, respectively.

*D*—H⋯*A*	*D*—H	H⋯*A*	*D*⋯*A*	*D*—H⋯*A*
C21—H21*B*⋯*Cg*5^i^	0.98	2.95	3.686 (3)	133
C22—H22*C*⋯*Cg*3^ii^	0.98	2.85	3.678 (2)	143
C32—H32⋯*Cg*5^iii^	0.95	2.87	3.518 (2)	126
C45—H45*A*⋯*Cg*1^ii^	0.98	2.89	3.820 (2)	160
O5—H45*C*⋯C45^iv^	0.98	2.69	3.336 (3)	123
O9—H34*B*⋯C34^iii^	0.98	2.77	3.177 (3)	106

Symmetry codes: (i) 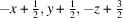; (ii) 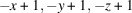; (iii) 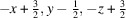; (iv) 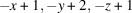.

**Table 2:** Experimental details

Crystal data	
Chemical formula	C_36_H_33_NO_6_S_3_
*M*_r_	671.81
Crystal system, space group	Monoclinic, *P*2_1_/*n*
Temperature (K)	100
*a*, *b*, *c* (Å)	15.5226 (2), 7.7010 (1), 26.7200 (3)
β (°)	91.204 (1)
*V* (Å^3^)	3193.39 (7)
*Z*	4
Radiation type	Cu *K*α
μ (mm^−1^)	2.53
Crystal size (mm)	0.21 × 0.16 × 0.06

Data collection	
Diffractometer	XtaLAB Synergy, Dualflex, HyPix
Absorption correction	Multi-scan (*CrysAlis PRO*; Rigaku OD, 2023 ▸)
*T*_min_, *T*_max_	0.743, 1.000
No. of measured, independent and observed [*I* > 2σ(*I*)] reflections	22361, 6361, 5681
*R*_int_	0.033
(sin θ/λ)_max_ (Å^−1^)	0.632

Refinement	
*R*[*F*^2^ > 2σ(*F*^2^)], *wR*(*F*^2^), *S*	0.041, 0.111, 1.02
No. of reflections	6361
No. of parameters	421
No. of restraints	3
H-atom treatment	H-atom parameters constrained
Δρ_max_, Δρ_min_ (e Å^−3^)	0.94, −0.42

Computer programs: *CrysAlis PRO* (Rigaku OD, 2023 ▸), *SHELXT2018/2* (Sheldrick, 2015*a* ▸), *SHELXL2018/3* (Sheldrick, 2015*b* ▸) and *OLEX2* (Dolomanov *et al.*, 2009 ▸).

## supplementary crystallographic information

### Tris[4-(3,4-dimethoxythiophen-2-yl)phenyl]amine Crystal data

**Table:**

C_36_H_33_NO_6_S_3_	*F*(000) = 1408
*M**_r_* = 671.81	*D*_x_ = 1.397 Mg m^−^^3^
Monoclinic, *P*2_1_/*n*	Cu *K*α radiation, λ = 1.54184 Å
*a* = 15.5226 (2) Å	Cell parameters from 14178 reflections
*b* = 7.7010 (1) Å	θ = 3.3–76.6°
*c* = 26.7200 (3) Å	µ = 2.53 mm^−^^1^
β = 91.204 (1)°	*T* = 100 K
*V* = 3193.39 (7) Å^3^	Block, colourless
*Z* = 4	0.21 × 0.16 × 0.06 mm

### Tris[4-(3,4-dimethoxythiophen-2-yl)phenyl]amine Data collection

**Table:**

XtaLAB Synergy, Dualflex, HyPix diffractometer	6361 independent reflections
Radiation source: micro-focus sealed X-ray tube, PhotonJet (Cu) X-ray Source	5681 reflections with *I* > 2σ(*I*)
Mirror monochromator	*R*_int_ = 0.033
Detector resolution: 10.0000 pixels mm^-1^	θ_max_ = 77.2°, θ_min_ = 3.3°
ω scans	*h* = −18→13
Absorption correction: multi-scan (CrysAlisPro; Rigaku OD, 2023)	*k* = −8→9
*T*_min_ = 0.743, *T*_max_ = 1.000	*l* = −33→33
22361 measured reflections	

### Tris[4-(3,4-dimethoxythiophen-2-yl)phenyl]amine Refinement

**Table:**

Refinement on *F*^2^	Primary atom site location: dual
Least-squares matrix: full	Hydrogen site location: inferred from neighbouring sites
*R*[*F*^2^ > 2σ(*F*^2^)] = 0.041	H-atom parameters constrained
*wR*(*F*^2^) = 0.111	*w* = 1/[σ^2^(*F*_o_^2^) + (0.0509*P*)^2^ + 3.2582*P*] where *P* = (*F*_o_^2^ + 2*F*_c_^2^)/3
*S* = 1.02	(Δ/σ)_max_ = 0.001
6361 reflections	Δρ_max_ = 0.94 e Å^−^^3^
421 parameters	Δρ_min_ = −0.42 e Å^−^^3^
3 restraints	

### Tris[4-(3,4-dimethoxythiophen-2-yl)phenyl]amine Special details

**Table:**

Geometry. All esds (except the esd in the dihedral angle between two l.s. planes) are estimated using the full covariance matrix. The cell esds are taken into account individually in the estimation of esds in distances, angles and torsion angles; correlations between esds in cell parameters are only used when they are defined by crystal symmetry. An approximate (isotropic) treatment of cell esds is used for estimating esds involving l.s. planes.
Refinement. 1. Fixed Uiso At 1.2 times of: All C(H) groups At 1.5 times of: All C(H,H,H) groups 2. Rigid body (RIGU) restrains C25, O7 with sigma for 1-2 distances of 0.004 and sigma for 1-3 distances of 0.004 3.a Aromatic/amide H refined with riding coordinates: C11(H11), C16(H16), C17(H17), C19(H19), C20(H20), C23(H23), C28(H28), C29(H29), C31(H31), C32(H32), C35(H35), C40(H40), C41(H41), C43(H43), C44(H44) 3.b Idealised Me refined as rotating group: C21(H21A,H21B,H21C), C22(H22A,H22B,H22C), C33(H33A,H33B,H33C), C34(H34A,H34B, H34C), C45(H45A,H45B,H45C), C46(H46A,H46B,H46C)

### Tris[4-(3,4-dimethoxythiophen-2-yl)phenyl]amine Fractional atomic coordinates and isotropic or equivalent isotropic displacement parameters (Å^2^)

**Table:**

	*x*	*y*	*z*	*U*_iso_*/*U*_eq_	
S1	0.15454 (3)	0.61897 (6)	0.74361 (2)	0.02413 (12)	
S2	0.88651 (3)	0.67913 (7)	0.84372 (2)	0.02604 (13)	
S3	0.65454 (3)	0.69538 (7)	0.42247 (2)	0.02619 (13)	
O4	−0.01202 (9)	0.8147 (2)	0.64844 (6)	0.0328 (4)	
O5	0.15437 (8)	0.84512 (19)	0.61326 (5)	0.0249 (3)	
O6	0.80763 (10)	0.6858 (2)	0.98068 (5)	0.0340 (4)	
O7	0.66877 (9)	0.7054 (2)	0.91195 (5)	0.0268 (3)	
O8	0.89878 (9)	0.7417 (2)	0.39631 (6)	0.0321 (3)	
O9	0.86873 (8)	0.70643 (19)	0.49969 (5)	0.0267 (3)	
N10	0.56623 (9)	0.6838 (2)	0.66955 (6)	0.0187 (3)	
C11	0.05196 (12)	0.6737 (3)	0.72270 (8)	0.0272 (4)	
H11	0.000890	0.652459	0.740695	0.033*	
C12	0.05418 (12)	0.7512 (3)	0.67704 (8)	0.0253 (4)	
C13	0.13939 (12)	0.7672 (3)	0.65852 (7)	0.0225 (4)	
C14	0.20158 (12)	0.7025 (2)	0.69029 (7)	0.0204 (4)	
C15	0.29556 (11)	0.6979 (2)	0.68452 (7)	0.0190 (4)	
C16	0.33683 (12)	0.8166 (2)	0.65342 (7)	0.0198 (4)	
H16	0.303730	0.900860	0.635563	0.024*	
C17	0.42564 (11)	0.8124 (2)	0.64839 (7)	0.0196 (4)	
H17	0.452921	0.894621	0.627473	0.024*	
C18	0.47490 (11)	0.6885 (2)	0.67382 (7)	0.0179 (4)	
C19	0.43444 (12)	0.5700 (3)	0.70489 (7)	0.0219 (4)	
H19	0.467624	0.484914	0.722364	0.026*	
C20	0.34577 (12)	0.5757 (3)	0.71040 (7)	0.0221 (4)	
H20	0.318898	0.495381	0.732090	0.027*	
C21	−0.09498 (13)	0.8072 (3)	0.67058 (10)	0.0395 (6)	
H21A	−0.137225	0.866666	0.648867	0.059*	
H21B	−0.092517	0.864110	0.703379	0.059*	
H21C	−0.112074	0.685583	0.674668	0.059*	
C22	0.15958 (17)	0.7259 (3)	0.57280 (9)	0.0398 (5)	
H22A	0.171514	0.789302	0.541908	0.060*	
H22B	0.104772	0.663664	0.568901	0.060*	
H22C	0.206030	0.642531	0.579691	0.060*	
C23	0.90087 (14)	0.6775 (3)	0.90789 (8)	0.0299 (4)	
H23	0.955181	0.669282	0.924775	0.036*	
C24	0.82440 (13)	0.6896 (3)	0.93090 (7)	0.0244 (4)	
C25	0.75199 (12)	0.7020 (2)	0.89652 (7)	0.0233 (4)	
C26	0.77498 (12)	0.6958 (2)	0.84772 (7)	0.0228 (4)	
C27	0.71990 (12)	0.6918 (2)	0.80234 (7)	0.0200 (4)	
C28	0.63873 (12)	0.7722 (3)	0.80027 (7)	0.0215 (4)	
H28	0.617920	0.829520	0.829087	0.026*	
C29	0.58852 (11)	0.7691 (2)	0.75675 (7)	0.0203 (4)	
H29	0.533869	0.824701	0.756097	0.024*	
C30	0.61729 (11)	0.6852 (2)	0.71385 (7)	0.0176 (4)	
C31	0.69720 (11)	0.6013 (2)	0.71623 (7)	0.0201 (4)	
H31	0.717290	0.540505	0.687819	0.024*	
C32	0.74708 (11)	0.6062 (3)	0.75967 (7)	0.0214 (4)	
H32	0.801441	0.549442	0.760393	0.026*	
C33	0.88133 (17)	0.6453 (4)	1.01196 (9)	0.0425 (6)	
H33A	0.904878	0.532511	1.002132	0.064*	
H33B	0.864043	0.640395	1.046997	0.064*	
H33C	0.925342	0.735222	1.008088	0.064*	
C34	0.64284 (15)	0.8624 (3)	0.93519 (8)	0.0350 (5)	
H34A	0.648322	0.958819	0.911527	0.053*	
H34B	0.679611	0.884297	0.964782	0.053*	
H34C	0.582716	0.852390	0.945285	0.053*	
C35	0.74226 (14)	0.7185 (3)	0.38445 (7)	0.0276 (4)	
H35	0.739170	0.723729	0.348938	0.033*	
C36	0.81654 (13)	0.7282 (3)	0.41233 (8)	0.0246 (4)	
C37	0.80256 (12)	0.7177 (2)	0.46504 (7)	0.0217 (4)	
C38	0.71760 (12)	0.7016 (2)	0.47683 (7)	0.0203 (4)	
C39	0.67883 (11)	0.6946 (2)	0.52628 (7)	0.0191 (4)	
C40	0.71954 (11)	0.7724 (2)	0.56792 (7)	0.0205 (4)	
H40	0.773563	0.828183	0.564031	0.025*	
C41	0.68258 (11)	0.7693 (2)	0.61449 (7)	0.0190 (4)	
H41	0.711357	0.823785	0.642015	0.023*	
C42	0.60348 (11)	0.6873 (2)	0.62174 (7)	0.0175 (4)	
C43	0.56175 (11)	0.6112 (3)	0.58047 (7)	0.0209 (4)	
H43	0.507540	0.556146	0.584383	0.025*	
C44	0.59910 (12)	0.6156 (3)	0.53377 (7)	0.0213 (4)	
H44	0.569634	0.563537	0.506108	0.026*	
C45	0.90692 (16)	0.7684 (3)	0.34378 (8)	0.0382 (5)	
H45A	0.890781	0.661829	0.325859	0.057*	
H45B	0.966713	0.798344	0.336488	0.057*	
H45C	0.868842	0.863281	0.332921	0.057*	
C46	0.91999 (14)	0.8600 (3)	0.50474 (9)	0.0358 (5)	
H46A	0.943976	0.890115	0.472241	0.054*	
H46B	0.967067	0.839203	0.529020	0.054*	
H46C	0.884058	0.955773	0.516431	0.054*	

### Tris[4-(3,4-dimethoxythiophen-2-yl)phenyl]amine Atomic displacement parameters (Å^2^)

**Table:**

	*U*^11^	*U*^22^	*U*^33^	*U*^12^	*U*^13^	*U*^23^
S1	0.0224 (2)	0.0272 (2)	0.0230 (2)	−0.00434 (18)	0.00633 (17)	−0.00136 (18)
S2	0.0185 (2)	0.0349 (3)	0.0245 (2)	−0.00048 (18)	−0.00314 (17)	0.00117 (19)
S3	0.0242 (2)	0.0361 (3)	0.0182 (2)	−0.00494 (19)	−0.00025 (18)	−0.00181 (19)
O4	0.0132 (6)	0.0371 (8)	0.0480 (9)	0.0029 (6)	0.0003 (6)	−0.0004 (7)
O5	0.0197 (6)	0.0288 (7)	0.0264 (7)	0.0023 (5)	0.0011 (5)	0.0050 (6)
O6	0.0402 (9)	0.0420 (9)	0.0196 (7)	0.0003 (7)	−0.0071 (6)	0.0000 (6)
O7	0.0210 (7)	0.0417 (8)	0.0178 (6)	0.0034 (6)	0.0034 (5)	0.0005 (6)
O8	0.0291 (7)	0.0366 (8)	0.0312 (8)	−0.0035 (6)	0.0155 (6)	−0.0029 (6)
O9	0.0185 (6)	0.0320 (8)	0.0295 (7)	−0.0016 (5)	−0.0001 (5)	−0.0009 (6)
N10	0.0128 (7)	0.0260 (8)	0.0172 (7)	−0.0002 (6)	−0.0005 (6)	0.0001 (6)
C11	0.0190 (9)	0.0273 (10)	0.0359 (11)	−0.0046 (8)	0.0102 (8)	−0.0074 (9)
C12	0.0152 (9)	0.0239 (10)	0.0369 (11)	−0.0006 (7)	0.0026 (8)	−0.0059 (8)
C13	0.0172 (9)	0.0222 (9)	0.0282 (10)	−0.0004 (7)	0.0029 (7)	−0.0021 (8)
C14	0.0182 (9)	0.0208 (9)	0.0223 (9)	−0.0025 (7)	0.0034 (7)	−0.0015 (7)
C15	0.0172 (9)	0.0218 (9)	0.0179 (8)	−0.0010 (7)	0.0010 (7)	−0.0023 (7)
C16	0.0178 (9)	0.0219 (9)	0.0197 (9)	0.0015 (7)	−0.0018 (7)	0.0016 (7)
C17	0.0174 (8)	0.0222 (9)	0.0191 (8)	−0.0022 (7)	−0.0002 (7)	0.0021 (7)
C18	0.0130 (8)	0.0231 (9)	0.0176 (8)	−0.0004 (7)	−0.0013 (6)	−0.0023 (7)
C19	0.0194 (9)	0.0233 (9)	0.0228 (9)	0.0008 (7)	−0.0019 (7)	0.0034 (7)
C20	0.0202 (9)	0.0241 (9)	0.0220 (9)	−0.0028 (7)	0.0013 (7)	0.0025 (7)
C21	0.0132 (9)	0.0449 (14)	0.0606 (16)	−0.0004 (9)	0.0051 (9)	−0.0135 (12)
C22	0.0530 (14)	0.0392 (13)	0.0273 (11)	−0.0008 (11)	−0.0006 (10)	0.0005 (10)
C23	0.0282 (10)	0.0326 (11)	0.0283 (10)	−0.0029 (8)	−0.0100 (8)	0.0008 (9)
C24	0.0306 (10)	0.0229 (9)	0.0196 (9)	−0.0015 (8)	−0.0054 (8)	−0.0003 (7)
C25	0.0229 (9)	0.0217 (9)	0.0253 (9)	−0.0017 (7)	−0.0010 (7)	−0.0007 (7)
C26	0.0233 (9)	0.0205 (9)	0.0242 (9)	−0.0013 (7)	−0.0044 (7)	0.0012 (7)
C27	0.0199 (9)	0.0205 (9)	0.0195 (9)	−0.0037 (7)	−0.0040 (7)	0.0041 (7)
C28	0.0226 (9)	0.0236 (9)	0.0182 (8)	−0.0025 (7)	0.0001 (7)	−0.0014 (7)
C29	0.0166 (8)	0.0232 (9)	0.0211 (9)	0.0013 (7)	0.0002 (7)	0.0014 (7)
C30	0.0146 (8)	0.0201 (9)	0.0181 (8)	−0.0018 (7)	−0.0021 (6)	0.0023 (7)
C31	0.0174 (8)	0.0219 (9)	0.0209 (9)	0.0011 (7)	−0.0004 (7)	−0.0007 (7)
C32	0.0163 (8)	0.0229 (9)	0.0249 (9)	0.0010 (7)	−0.0032 (7)	0.0030 (7)
C33	0.0535 (15)	0.0492 (15)	0.0240 (11)	−0.0042 (12)	−0.0190 (10)	0.0024 (10)
C34	0.0400 (12)	0.0367 (12)	0.0286 (11)	0.0050 (10)	0.0034 (9)	−0.0030 (9)
C35	0.0344 (11)	0.0284 (10)	0.0202 (9)	−0.0049 (8)	0.0058 (8)	−0.0013 (8)
C36	0.0266 (10)	0.0207 (9)	0.0267 (10)	−0.0023 (8)	0.0089 (8)	−0.0023 (8)
C37	0.0205 (9)	0.0204 (9)	0.0243 (9)	−0.0005 (7)	0.0029 (7)	−0.0008 (7)
C38	0.0206 (9)	0.0217 (9)	0.0185 (9)	−0.0021 (7)	0.0000 (7)	−0.0012 (7)
C39	0.0176 (8)	0.0210 (9)	0.0189 (9)	0.0014 (7)	0.0005 (7)	−0.0001 (7)
C40	0.0142 (8)	0.0238 (9)	0.0234 (9)	−0.0018 (7)	−0.0005 (7)	−0.0001 (7)
C41	0.0160 (8)	0.0213 (9)	0.0195 (9)	−0.0011 (7)	−0.0017 (7)	−0.0012 (7)
C42	0.0150 (8)	0.0210 (9)	0.0166 (8)	0.0026 (7)	−0.0008 (6)	0.0012 (7)
C43	0.0154 (8)	0.0263 (10)	0.0211 (9)	−0.0038 (7)	−0.0008 (7)	−0.0001 (7)
C44	0.0191 (8)	0.0260 (10)	0.0188 (9)	−0.0036 (7)	−0.0027 (7)	−0.0012 (7)
C45	0.0472 (13)	0.0383 (13)	0.0300 (11)	−0.0083 (11)	0.0221 (10)	−0.0078 (10)
C46	0.0248 (10)	0.0436 (13)	0.0391 (12)	−0.0110 (9)	0.0030 (9)	−0.0059 (10)

### Tris[4-(3,4-dimethoxythiophen-2-yl)phenyl]amine Geometric parameters (Å, º)

**Table:**

S1—C11	1.729 (2)	C15—C20	1.396 (3)
S1—C14	1.7381 (19)	C16—C17	1.388 (3)
S2—C23	1.724 (2)	C17—C18	1.391 (3)
S2—C26	1.742 (2)	C18—C19	1.392 (3)
S3—C35	1.725 (2)	C19—C20	1.388 (3)
S3—C38	1.7351 (19)	C23—C24	1.351 (3)
O4—C12	1.359 (2)	C24—C25	1.440 (3)
O4—C21	1.430 (3)	C25—C26	1.360 (3)
O5—C13	1.374 (2)	C26—C27	1.469 (3)
O5—C22	1.422 (3)	C27—C28	1.404 (3)
O6—C24	1.361 (2)	C27—C32	1.390 (3)
O6—C33	1.437 (3)	C28—C29	1.386 (3)
O7—C25	1.364 (2)	C29—C30	1.397 (3)
O7—C34	1.421 (3)	C30—C31	1.399 (2)
O8—C36	1.359 (2)	C31—C32	1.382 (3)
O8—C45	1.427 (3)	C35—C36	1.362 (3)
O9—C37	1.371 (2)	C36—C37	1.432 (3)
O9—C46	1.430 (3)	C37—C38	1.368 (3)
N10—C18	1.425 (2)	C38—C39	1.464 (3)
N10—C30	1.411 (2)	C39—C40	1.402 (3)
N10—C42	1.414 (2)	C39—C44	1.397 (3)
C11—C12	1.359 (3)	C40—C41	1.381 (3)
C12—C13	1.427 (3)	C41—C42	1.398 (3)
C13—C14	1.366 (3)	C42—C43	1.396 (3)
C14—C15	1.471 (2)	C43—C44	1.387 (3)
C15—C16	1.400 (3)		
			
S1^i^···H45A	2.93	C26^iv^···H41	2.89
O5^ii^···H45C	2.69	C27^vii^···H31	2.87
O6^iii^···H22A	2.71	C28^vii^···H32	2.85
O9^iv^···C34	3.177 (3)	C29^vii^···H32	2.80
C11^v^···H19	2.83	C36^i^···H22C	2.88
C13^ii^···H45C	2.86	C46^viii^···C46	3.303 (3)
C17^vi^···H33B	2.88	H21A^ix^···H41	2.38
C24^iv^···C40	3.285 (3)	H44^i^···H44	2.39
C26^vii^···H31	2.82		
			
C11—S1—C14	92.56 (9)	O7—C25—C24	122.67 (18)
C23—S2—C26	92.71 (10)	C26—C25—O7	124.03 (17)
C35—S3—C38	92.97 (10)	C26—C25—C24	113.10 (18)
C12—O4—C21	115.45 (18)	C25—C26—S2	110.04 (14)
C13—O5—C22	113.62 (16)	C25—C26—C27	129.21 (18)
C24—O6—C33	114.01 (18)	C27—C26—S2	120.65 (15)
C25—O7—C34	115.28 (16)	C28—C27—C26	121.98 (17)
C36—O8—C45	115.09 (18)	C32—C27—C26	120.36 (17)
C37—O9—C46	114.76 (16)	C32—C27—C28	117.67 (16)
C30—N10—C18	118.36 (14)	C29—C28—C27	120.92 (17)
C30—N10—C42	121.67 (14)	C28—C29—C30	120.82 (17)
C42—N10—C18	119.88 (14)	C29—C30—N10	120.56 (16)
C12—C11—S1	110.90 (14)	C29—C30—C31	118.34 (16)
O4—C12—C11	129.03 (18)	C31—C30—N10	121.10 (16)
O4—C12—C13	117.98 (19)	C32—C31—C30	120.40 (17)
C11—C12—C13	112.98 (18)	C31—C32—C27	121.82 (17)
O5—C13—C12	121.20 (17)	C36—C35—S3	110.69 (15)
C14—C13—O5	125.14 (17)	O8—C36—C35	128.47 (19)
C14—C13—C12	113.64 (18)	O8—C36—C37	118.49 (18)
C13—C14—S1	109.92 (14)	C35—C36—C37	113.01 (17)
C13—C14—C15	129.30 (17)	O9—C37—C36	122.75 (17)
C15—C14—S1	120.78 (14)	C38—C37—O9	123.42 (17)
C16—C15—C14	120.92 (16)	C38—C37—C36	113.49 (17)
C20—C15—C14	120.61 (17)	C37—C38—S3	109.83 (14)
C20—C15—C16	118.47 (17)	C37—C38—C39	128.88 (17)
C17—C16—C15	120.67 (17)	C39—C38—S3	121.27 (14)
C16—C17—C18	120.36 (17)	C40—C39—C38	120.88 (16)
C17—C18—N10	121.06 (16)	C44—C39—C38	121.83 (17)
C17—C18—C19	119.38 (16)	C44—C39—C40	117.26 (16)
C19—C18—N10	119.54 (16)	C41—C40—C39	121.25 (17)
C20—C19—C18	120.22 (17)	C40—C41—C42	121.01 (17)
C19—C20—C15	120.89 (17)	C41—C42—N10	120.73 (16)
C24—C23—S2	110.83 (15)	C43—C42—N10	120.93 (16)
O6—C24—C25	117.53 (18)	C43—C42—C41	118.33 (16)
C23—C24—O6	129.13 (18)	C44—C43—C42	120.28 (17)
C23—C24—C25	113.30 (18)	C43—C44—C39	121.86 (17)

Symmetry codes: (i) −*x*+1, −*y*+1, −*z*+1; (ii) −*x*+1, −*y*+2, −*z*+1; (iii) *x*−1/2, −*y*+1/2, *z*−1/2; (iv) −*x*+3/2, *y*−1/2, −*z*+3/2; (v) −*x*+1/2, *y*+1/2, −*z*+3/2; (vi) *x*−3/2, −*y*+1/2, *z*−3/2; (vii) −*x*+3/2, *y*+1/2, −*z*+3/2; (viii) −*x*+2, −*y*+2, −*z*+1; (ix) *x*−1, *y*, *z*.

### Tris[4-(3,4-dimethoxythiophen-2-yl)phenyl]amine Hydrogen-bond geometry (Å, º)

*Cg*1, *Cg*3 and *Cg*5 are the centroids of the S1/C11–C14,S3/C35–C38 and C27–C32 rings, respectively.

**Table:**

*D*—H···*A*	*D*—H	H···*A*	*D*···*A*	*D*—H···*A*
C21—H21*B*···*Cg*5^v^	0.98	2.95	3.686 (3)	133
C22—H22*C*···*Cg*3^i^	0.98	2.85	3.678 (2)	143
C32—H32···*Cg*5^iv^	0.95	2.87	3.518 (2)	126
C41—H41···*Cg*2^vii^	0.95	2.96	3.2346 (17)	98
C45—H45*A*···*Cg*1^i^	0.98	2.89	3.820 (2)	160
S1—H20···C20^v^				
O5—H45*C*···C45^ii^	0.98	2.69	3.336 (3)	123
O9—H34*B*···C34^iv^	0.98	2.77	3.177 (3)	106

Symmetry codes: (i) −*x*+1, −*y*+1, −*z*+1; (ii) −*x*+1, −*y*+2, −*z*+1; (iv) −*x*+3/2, *y*−1/2, −*z*+3/2; (v) −*x*+1/2, *y*+1/2, −*z*+3/2; (vii) −*x*+3/2, *y*+1/2, −*z*+3/2.

## References

[bb1] Adelizzi, B., Rösch, A. T., van Rijen, D. J., Martire, R. S., Esiner, S., Lutz, M., Palmans, A. R. A. & Meijer, E. W. (2019). *Helv. Chim. Acta* **102**, e1900065.

[bb2] Dolomanov, O. V., Bourhis, L. J., Gildea, R. J., Howard, J. A. K. & Puschmann, H. (2009). *J. Appl. Cryst.* **42**, 339–341.

[bb3] Golba, S., Starczewska, O. & Idzik, K. (2015). *Des. Monomers Polym.* **18**, 770–779.

[bb21] Groom, C. R., Bruno, I. J., Lightfoot, M. P. & Ward, S. C. (2016). *Acta Cryst*. B**72**, 171–179. 10.1107/S2052520616003954 PMC4822653 27048719

[bb5] Lambert, C. & Nöll, G. (1999). *J. Am. Chem. Soc.* **121**, 8434–8442.

[bb6] McKinnon, J. J., Jayatilaka, D. & Spackman, M. A. (2007). *Chem. Commun.* pp. 3814–3816. 10.1039/b704980c 18217656

[bb22] Parthasarathy, V., Fery–Forgues, S., Campioli, E., Recher, G., Terenziani, F. & Blanchard-Desce, M. (2011). *Small* **7**, 3219–3229. 10.1002/smll.201100726 21972222

[bb7] Peng, Z. (2025*a*). *CSD Communication* (refcode ILIWAF, CCDC 2412701). CCDC, Cambridge, England.

[bb8] Peng, Z. (2025*b*). *CSD Communication* (refcode ILIWEJ, CCDC 2412703). CCDC, Cambridge, England.

[bb9] Rigaku OD (2023). *CrysAlis PRO*. Rigaku Oxford Diffraction, Yarnton, England.

[bb10] Sato, K., Yano, M., Furuichi, M., Shiomi, D., Takui, T., Abe, K., Itoh, K., Higuchi, A., Katsuma, K. & Shirota, Y. (1997). *J. Am. Chem. Soc.* **119**, 6607–6613.

[bb11] Sheldrick, G. M. (2015*a*). *Acta Cryst.* A**71**, 3–8.

[bb12] Sheldrick, G. M. (2015*b*). *Acta Cryst.* C**71**, 3–8.

[bb13] Spackman, M. A. & Jayatilaka, D. (2009). *CrystEngComm* **11**, 19–32.

[bb14] Spackman, M. A. & McKinnon, J. J. (2002). *CrystEngComm* **4**, 378–392.

[bb15] Spackman, P. R., Turner, M. J., McKinnon, J. J., Wolff, S. K., Grimwood, D. J., Jayatilaka, D. & Spackman, M. A. (2021). *J. Appl. Cryst.* **54**, 1006–1011. 10.1107/S1600576721002910 PMC8202033 34188619

[bb20] Thelakkat, M. (2002). *Macromol. Mater. Eng.* **287**, 442–461.

[bb16] Wang, Q., He, Z., Wild, A., Wu, H., Cao, Y., Schubert, U. S., Chui, C. H. & Wong, W. Y. (2011). *Chem. ? An. Asia. J.* **6**, 1766–1777. 10.1002/asia.201100111 21656689

[bb17] Yano, M., Inada, Y., Hayashi, Y., Nakai, M., Mitsudo, K. & Kashiwagi, Y. (2022). *Dyes Pigments* **197**, 109929.

[bb18] Yen, H.-J. & Liou, G.-S. (2012). *Polym. Chem.* **3**, 255–264.

[bb19] Yuan, G., Lv, C., Liang, J., Zhong, X., Li, Y., He, J., Zhao, A., Li, L., Shao, Y., Zhang, X., Wang, S., Cheng, Y. & He, H. (2021). *Adv. Funct. Mater.* **31**, 2104026.

